# Temporal dynamics of the tick *Ixodes ricinus* in northern Europe: epidemiological implications

**DOI:** 10.1186/s13071-017-2112-x

**Published:** 2017-03-31

**Authors:** Claire Cayol, Esa Koskela, Tapio Mappes, Anja Siukkola, Eva R. Kallio

**Affiliations:** Department of Biological and Environmental Science, University of Jyväskylä, P.O. Box 35, FI-40014 Jyväskylä, Finland

**Keywords:** *Ixodes ricinus*, Rodent host, Seasonality, Public health, Population dynamics

## Abstract

**Background:**

Tick-borne pathogens pose an increasing threat to human and veterinary health across the northern hemisphere. While the seasonal activity of ticks is largely determined by climatic conditions, host-population dynamics are also likely to affect tick abundance. Consequently, abundance fluctuations of rodents in northern Europe are expected to be translated into tick dynamics, and can hence potentially affect the circulation of tick-borne pathogens. We quantified and explained the temporal dynamics of the tick *Ixodes ricinus* in the northernmost part of its European geographical range, by estimating (i) abundance in vegetation and (ii) infestation load in the most common rodent species in the study area, the bank vole *Myodes glareolus*.

**Results:**

*Ixodes ricinus* nymphs and adult females, the life stages responsible for the most of tick bites in humans, peaked in May-June and August-September. Larvae and nymphs were simultaneously active in June and abundance of questing larvae and nymphs in the vegetation showed a positive association with bank vole abundance. Moreover, infesting larvae and nymphs were aggregated on bank voles, and the infestation of bank voles with *I. ricinus* larvae and nymphs was positively associated with bank vole abundance.

**Conclusion:**

Our results indicate early summer and early autumn as periods of increased risk for humans to encounter *I. ricinus* ticks in boreal urban forests and suggest a 2 years life-cycle for *I. ricinus* with two cohorts of ticks during the same year. Moreover, we identified a simultaneous activity of larvae and nymphs which allows co-feeding on the rodent host, which in turn supports the transmission of several important zoonotic tick-borne pathogens. Finally, we showed that a high density of the rodent host may enhance the risk that ticks and, potentially, tick-borne pathogens pose to human health.

**Electronic supplementary material:**

The online version of this article (doi:10.1186/s13071-017-2112-x) contains supplementary material, which is available to authorized users.

## Background

Tick-borne pathogens are a growing burden for European public health policies [1–3]. The current observed increase in tick-borne disease incidence in Europe may be explained by the geographical expansion of *Ixodes ricinus*, the growing share of space between humans and wild animals, and the improvement of diagnostics tools [4–6]. The epidemiology of tick-borne zoonoses, such as Lyme borreliosis, anaplasmosis or tick-borne encephalitis (TBE), depends on tick abundance and population dynamics, infection prevalence within the tick population, and land use that may affect human exposure to ticks [7, 8]. In order to predict the risks that tick-borne diseases pose to humans, an assessment of factors underlying the temporal variation of tick abundance is necessary.

The abundance of *I. ricinus* varies in time and space and is highly dependent on environmental conditions, including habitat quality, host availability, and abiotic conditions [9–12]. In northern Fennoscandia, at the northernmost part of the European range of *I. ricinus*, abiotic conditions undergo extreme seasonal variation; there are 145 to 160 days of snow cover with short day-lengths, during which ticks are not active. This is followed by a quick elevation in temperature leading to a short summer with long day-lengths [13]. In these conditions, *I. ricinus* activity is likely to show distinctive seasonal patterns, which have not been characterized to date (but see [14] for southwest Finland).

*Ixodes ricinus* is dependent on vertebrate hosts to complete its life-cycle. Larvae typically feed on small vertebrates, such as rodents; nymphs, the more common biting stage for humans, parasitize mostly medium-sized mammals; and adults feed mainly on large hosts, such as deer [15, 16]. The population dynamics of ticks and rodents are expected to be linked: some studies have indicated delayed density dependence of questing nymphs on rodent abundance, suggesting that high rodent abundance provides augmented opportunities for successful larvae feeding and nymph development [17, 18]. The bank vole (*Myodes glareolus*) is a common rodent species throughout Europe [19]; this species is commonly infested by immature *I. ricinus* [16, 20, 21]. In northern Europe, vole population abundance shows both seasonality, driven by seasonal breeding, and multiannual density fluctuations shaped by predation, food availability and food quality [22–24]. These seasonal and multiannual density fluctuations are likely to be translated into the dynamics of ticks, and consequently, into the epidemiology of tick-borne pathogens. To date, there are few studies that have investigated the association between the dynamics of cyclic small rodents and ticks [25].

The bank vole is also an important reservoir host for many tick-borne pathogens, such as *Borrelia afzelii*, tick-borne encephalitis virus (TBEV) and *Babesia microti* [26, 27]. Typically, tick larvae acquire infections from an infected rodent host that has become infected while feeding infected nymph(s) [10]. Alternatively, larvae acquire infections *via* simultaneous feeding with infected nymphs without systemic infection of the host [28, 29]. Infectivity is transstadially maintained in the tick to the following life stage [30].

Here, we present results from a 4 years of longitudinal bank vole monitoring and tick sampling in central Finland at the northernmost part of the European range of *I. ricinus*, where abiotic conditions undergo extreme seasonal variation. Our primary aim is to characterize temporal dynamics and quantify the importance of host related factors and abiotic conditions on temporal dynamics of *I. ricinus*. We also aim to identify seasonal patterns that are relevant for tick-borne pathogen circulation, with the ultimate goal of providing information concerning the risk of tick-borne diseases in our study area.

## Methods

### Study area

Sampling took place monthly from May to October in 2012 − 2015 in four periurban forests in the Jyväskylä area in Central Finland: (Kylmänoro (62°13′36.220″, 25°45′1.739″); Jyskänlaakso (62°13′55.398″, 25°49′34.269″); Hämeenlahti (62°12′40.119″, 25°47′11.052″); and Sippulanniemi (62°11′9.019″, 25°44′58.147″) [31]. One trapping period within a month will be referred to as “session” in the following paragraphs. Forests were dominated by Scots pine (*Pinus sylvestris*) and silver birch (*Betula pendula*) or by spruce (*Picea abies*). The herbaceous stratum was typically composed of *Vaccinium myrtillus*, *V. vitis-idaea*, *Maianthemum bifolium*, *Linnaea borealis* and *Oxalis acetosella*.

### Tick dragging

Monthly tick dragging was performed during or within a few days of the vole trapping, using a 1 × 1 m cotton flannel flag sewed to a wooden rod [12]. The fabric was randomly dragged over the vegetation for 300 − 500 m per site around the rodent trapping transects and checked every 20−25 m for ticks, which were removed with tweezers and stored in alcohol at -20 °C. No dragging was performed during rain. In October 2014, due to early snow cover, dragging was not performed. Due to the duration, coverage and interval of the dragging (less than 30 min, 300–500 m^2^ once a month in each site) it is unlikely that the flag dragging affected the overall tick population abundance and it should not have interfered with the ticks parasitizing rodents in the area.

### Vole trapping and tick infestation on voles

As the active tick population consists in parasitizing, questing and resting ticks, sampling targeted questing ticks and parasitizing ticks on their rodent host. This latter buffers the effects of microclimate changes and rodent sampling, in particular, also buffers the effect of the patchy distribution of larvae [32].

Vole trapping was carried out with two lines of 10 Ugglan Special multiple-capture live traps (Grahnab Company, Sweden), positioned 10 − 15 m apart, located near to rodent burrows. Traps were prebaited for 1−3 nights with sunflower seeds (*Helianthus annuus*), after which traps were set with sunflower seeds (for food) and a piece of potato (for water) for two consecutive nights. Wood shavings were provided as bedding in wet or cold weather. Traps were checked once per day and trapped voles were handled and sampled before release close to their capture site. Bycatch of species other than voles, as well as recapture of the same individual during the same session, were released immediately on site.

All trapped voles were marked individually with electronic identification chips (microchip Trovan Unique™), which were injected subcutaneously at their first capture. During each capture, voles were identified, body mass was measured as a proxy for age (as in e.g. [33]), and sex and reproductive condition were recorded. The presence of fleas was recorded and all voles were examined for ticks, with special attention to the area around the ears and face. All ticks were removed with tweezers and stored in alcohol at -20 °C until further identification. All ticks - both those removed from rodents and those collected from vegetation - were identified to species level and life stage under a dissection microscope using morphological identification keys [34–36]. Species identification of seven ticks identified as *I. ricinus* and three as *I. trianguliceps* was further confirmed with PCR following a method described elsewhere [37]. Briefly, PCR targeted the mitochondrial 16S rRNA gene and the amplicons obtained were successfully sequenced for eight of the ten ticks. Thereafter sequence identity was determined by BLAST search against the NCBI Nucleotide database and the obtained sequences confirmed our morphological tick identification.

We assessed the overall bank vole population abundance by computing the overall minimum number of voles alive (MNA) at a given trapping session (t) as follows: total number of individuals caught at a given trapping session (t) summed with the total number of individuals marked when caught during subsequent sessions, but not caught at (t) [38].

We trapped 658 bank voles, an average of 1.53 times (range 1–6), for a total of 1007observations for which all variables described above were available. The minimum number of voles alive per session varied from 5 (in May 2013) to 120 individuals (in September 2014). Three other rodent species were bycaught, consisting of 52 observations of yellow-necked mouse (*Apodemus flavicollis*), one observation of field vole (*Microtus agrestis*), and two observations of house mouse (*Mus musculus*) (Additional file 1: Figure S3).

### Statistical analysis

#### Ticks in vegetation

We characterized the temporal activity of *I. ricinus* in the vegetation (i.e. collected by flagging), by examining tick questing activity separately for each life stage, i.e. larvae, nymphs, adults (males and females), in relation to the following variables: year (2012 − 2015), month (May-October), estimated bank vole abundance per given session (MNA), abundance of other life stages present during the same session (number of ticks/100 m^2^), and the abundance of previous tick life stages collected during the previous session (for larvae: adult, for nymph: larvae, for adults: nymphs). To further identify the effect of current climatic conditions on tick activity, we computed the mean daily saturation deficit (SatDef, in millimetres of mercury) during tick flagging days, based on daily average humidity (in percent) and daily average temperature (in °C) [9, 39, 40] recorded at the meteorological station of Nenäinniemi in Jyväskylä, located 0.72−3.7 km from the study sites (http://www.jyv-weather.info/index.php) (Additional file 1: Figure S1). SatDef was used as an explanatory variable rather than month, with which it showed collinearity. Thus, the second set of models included SatDef and its second-degree polynomial term SatDef^2^, MNA, current and previous tick abundances as described above. Furthermore, the abundance of nymphs and females pooled together was also modelled with two sets of models: the first one included vole abundance, month and year and the second one included year, vole abundance, SatDef and SatDef^2^.

Models were fitted using generalized linear mixed models (GLMM) with a negative binomial error distribution (with log-link function) and site was included as a random effect to control for potential pseudoreplication [41]. To take into account the variation in the distance flags were dragged, an offset term (log(distance flagged)/100) was introduced in the models. The model selection (provided in Additional file 1: Table S2) was an automated selection process starting from the full model and based on AICc (Akaike Information Criteria corrected for small sample size [42]), using dredge function in R software. We kept the most parsimonious model that lay within 2AICc difference from the best model fitted [42] (Additional file 1: Tables S1 and S2).

#### Ticks infesting voles

*Ixodes ricinus* infestation load on bank voles was examined separately for larvae and nymphs. We assessed whether tick infestation showed seasonality and/or between year variation and whether it was affected by individual host characteristics or by concomitant parasitism (by other tick stages, other tick species or fleas). For that purpose, we fitted a GLMM with a negative binomial error distribution to test the fixed effects of month, year, vole sex, body mass (centred value) and its second order polynomial term, presence of fleas, presence of other life stages of *I. ricinus* and *I. trianguliceps*, body mass*vole abundance (MNA) interaction term and body mass*sex interaction term. ‘Trapping site’ and ‘vole individual nested in the trapping site’ were included as random effects in the models. Model selection was performed as described before except that we utilized the function *drop1* in R software (Additional file 1: Tables S4 and S5).

All statistical analyses were performed with R version 3.2.3 (2015, The R Foundation for Statistical Computing), and using the packages *stats* (http://www.R-project.org/), *MASS* (https://cran.r-project.org/web/packages/MASS/index.html), *glmmADMB* (http://glmmadmb.r-forge.r-project.org/) and *MuMIn* (https://cran.r-project.org/web/packages/MuMIn/index.html).

## Results

### Ticks in vegetation

We sampled and identified 943 *I. ricinus* larvae, 867 nymphs, 239 (adult) females and 294 males from the vegetation. The mean abundance of *I. ricinus* per session and per area varied from 0 to 22.7 ticks/100 m^2^ when considering all tick life stages and from 0 to 6.25 ticks/100 m^2^ when taking into account only female adults and nymphs. Overall, the density of questing ticks collected from vegetation was 7.1/100 m^2^ (Additional file 1: Figure S2). The ratio between *I. ricinus* larvae, nymphs and adults was 3.5:3.3:2.0. In addition, one *I. trianguliceps* nymph was identified.

**Fig. 1:**
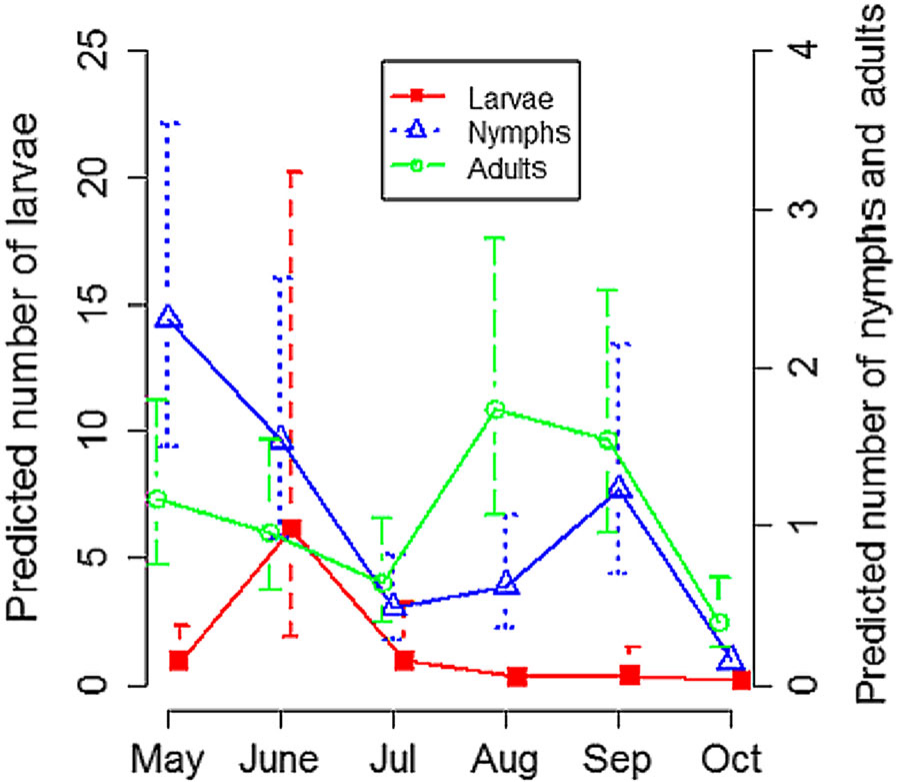
Predicted number ± standard error (SE) of larvae, nymphs and adults in 100 m^2^ of vegetation per month. Predictions are based on GLMM shown in Table 1

Models revealed unimodal questing patterns for larvae, which were mostly found in June. Conversely, a bimodal questing pattern was found for nymphs as well as nymph and females modelled together, with the highest abundances found in May-June and September, which therefore appears as the higher risk period for tick bites on humans. Questing adults (males and females) were more abundant in May-June and August-September, and their abundance varied with year, with the highest abundance found in 2015 (Table 1, Fig. 1).

**Table 1:** Selected best model for the abundance of tick questing in the vegetation with estimated coefficients (in log scale), explained by vole abundance, month (May taken as reference) and year (2012 as reference)

*Y = Larva abundance*	Estimate (SE)	*z*-value	*P*-value
Intercept	-1.424 (0.667)	-2.13	0.033
June	1.891 (0.742)	2.55	0.011
July	0.003 (0.778)	0.00	0.997
August	-1.100 (0.974)	-1.13	0.259
September	-0.987 (1.035)	-0.95	0.340
October	-1.707 (0.979)	-1.74	0.081
Vole abundance	0.028 (0.013)	2.14	0.032
Random effect: site	σ^2^ = 0.46 (SD = 0.68)		
Negative binomial dispersion parameter	0.38 (SE = 0.07)		
*Y = Nymph abundance*	Estimate (SE)	*z*-value	*P*-value
Intercept	0.193 (0.375)	0.52	0.607
June	-0.401 (0.275)	-1.46	0.145
July	-1.571 (0.313)	-5.02	<0.005
August	-1.312 (0.351)	-3.74	<0.005
September	-0.628 (0.390)	-1.61	0.107
October	-2.730 (0.404)	-6.76	<0.005
Vole abundance	0.013 (0.004)	2.99	0.003
Random effect: site	σ^2^ = 0.38 (SD = 0.62)		
Negative binomial dispersion parameter	2.88 (SE = 0.70)		
*Y = Adult (male + female) abundance*	Estimate (SE)	*z*-value	*P*-value
Intercept	-0.766 (0.393)	-1.95	0.051
June	-0.203 (0.226)	-0.90	0.368
July	-0.600 (0.238)	-2.52	0.012
August	0.395 (0.215)	1.84	0.066
September	0.279 (0.214)	1.30	0.192
October	-1.082 (0.292)	-3.71	<0.005
2013	0.288 (0.196)	1.47	0.142
2014	0.306 (0.202)	1.51	0.131
2015	0.923 (0.191)	4.82	<0.005
Random effect: site	σ^2^ = 0.39 (SD = 0.63)		
Negative binomial dispersion parameter	7.48 (SE = 2.81)		
*Y = Female + Nymph abundance*	Estimate (SE)	*z*-value	*P*-value
Intercept	0.514 (0.350)	1.47	0.142
June	-0.422 (0.227)	-1.86	0.063
July	-1.509 (0.258)	-5.84	<0.005
August	-1.021 (0.282)	-3.62	<0.005
September	-0.599 (0.324)	-1.85	0.064
October	-2.430 (0.323)	-7.53	<0.005
Vole abundance	0.012 (0.004)	3.04	0.002
Random effect: site	σ^2^ = 0.37 (SD = 0.60)		
Negative binomial dispersion parameter	4.42 (SE = 1.10)		

σ^2^ is the variance attributable to random effect. Number of observations: Total = 88; Site = 4

*Abbreviations*: *SD* standard deviation, *SE* standard error

**Fig. 2:**
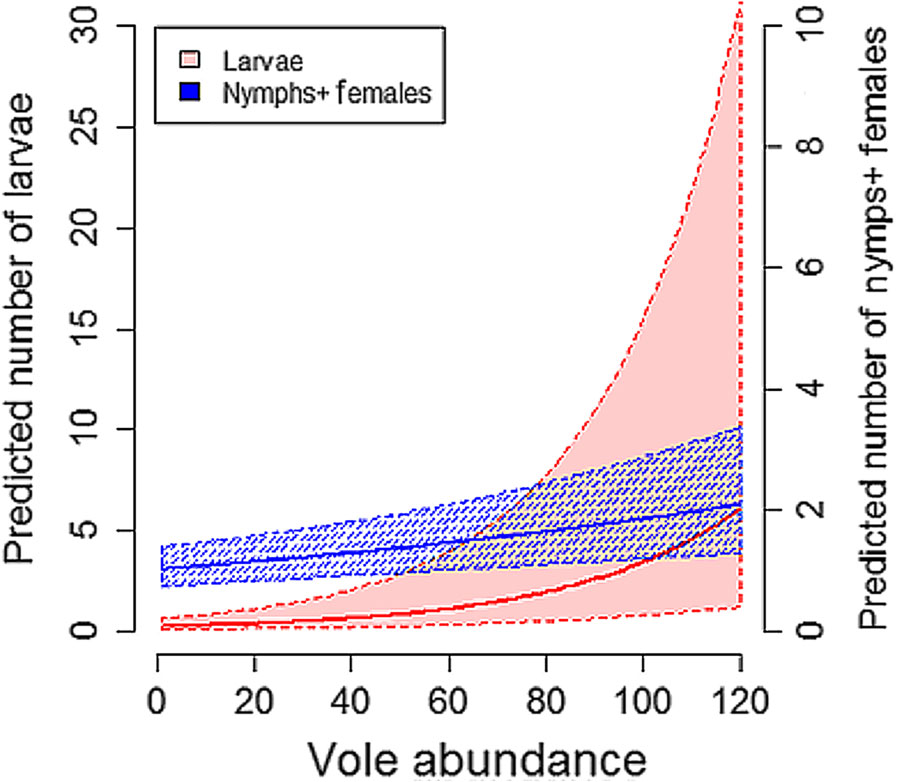
Predicted number ± standard error (SE) of larvae and pooled nymphs and females in 100 m^2^ of vegetation explained by vole abundance. Predictions are based on GLMM shown in Table 2

For any given month, the abundances of questing *I. ricinus* larvae and nymphs showed positive associations with vole abundance (Tables 1, 2; Fig. 2; Additional file 1: Figure S4). For each addition of one individual to the bank vole population, an increase of larvae abundance by approximately 3% and of nymph abundance by 1% was predicted (Fig. 2; Additional file 1: Figure S4).

**Table 2:** Selected best model for the abundance of ticks questing in the vegetation with estimated coefficients (in log scale), explained by the vole abundance, the amount of ticks in other stages in vegetation during the previous session and/or during the current session, and the saturation deficit (SatDef) and its second degree polynomial term (SatDef^2^)

*Y = Larva abundance*	Estimate (SE)	*z*-value	*P*-value
Intercept	-5.426 (1.002)	-5.41	<0.005
Vole abundance	0.029 (0.009)	3.11	0.002
Amount of adult ticks during the previous session	1.007 (0.308)	3.27	0.001
SatDef	0.969 (0.192)	5.03	<0.005
Random effect: site	σ^2^ = 4.59e^-06^ (SD = 0.002)		
Negative binomial dispersion parameter	0.34 (SE = 0.06)		
*Y = Nymph abundance*	Estimate (SE)	*z*-value	*P*-value
Intercept	-0.279 (0.233)	-1.20	0.232
Amount of adult ticks during the same session	0.381 (0.167)	2.28	0.023
Random effect: site	σ^2^ = 0.098 (SD = 0.31)		
Negative binomial dispersion parameter	1.12 (SE = 0.21)		
*Y = Adult (male + female) abundance*	Estimate (SE)	*z*-value	*P*-value
Intercept	-1.294 (0.461)	-2.81	0.005
SatDef	0.621 (0.239)	2.60	0.009
SatDef^2^	-0.098 (0.037)	-2.63	0.009
Amount of nymph during the same session	0.222 (0.075)	2.97	0.003
Amount of nymph during the previous session	-0.135 (0.071)	-1.91	0.056
Random effect: site	σ^2^ = 0.33 (SD = 0.57)		
Negative binomial dispersion parameter	3.04 (SE = 0.81)		
*Y = Female + nymph abundance*	Estimate (SE)	*z*-value	*P*-value
Intercept	0.011 (0.316)	0.04	0.971
Vole abundance	0.006 (0.003)	1.98	0.048
Random effect: site	σ^2^ = 0.27 (SD = 0.52)		
Negative binomial dispersion parameter	1.53 (SE = 0.29)		

σ^2^ is the variance attributable to random effect. Number of observations: Total = 88; Site = 4

*Abbreviations*: *SD* standard deviation, *SE* standard error

The abundance of questing larvae was positively associated with saturation deficit, while the abundance of questing adults showed a non-linear relationship with saturation deficit. The number of adults found in vegetation was positively associated with saturation deficit until an optimal value (3.16 mm Hg), after which the abundance of adult ticks was negatively affected by any further increase in saturation deficit. Nymph abundance was not associated with saturation deficit in the best model selected (Table 2).

We found a positive relationship between the number of questing larvae and the abundance of adults observed in the vegetation one session before. Nymph abundance increased with adult abundance during the same flagging session whereas adult abundance showed a negative relationship with nymph abundance during the previous session (Table 2).

### Ticks infesting voles

From bank voles, two tick species were identified: *I. trianguliceps*, the vole tick and *I. ricinus*. The proportion of infestation with either of these tick species was 75.8%. The ratio of *I. ricinus* larvae to nymphs found feeding on bank voles was 13:1. The total number of ticks sampled from voles was 3564, out of which 14 ticks could not be identified due to poor condition (Additional file 1: Table S3 and Figure S5).

**Fig. 3:**
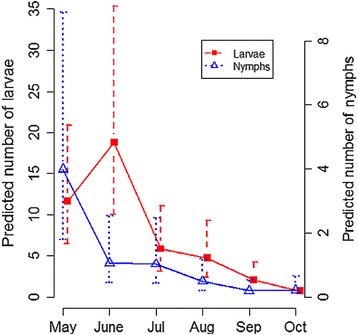
Predicted number ± standard error (SE) of larvae and nymphs on a vole per month. Predictions are based on GLMM shown in Tables 3 and 4

**Fig. 4:**
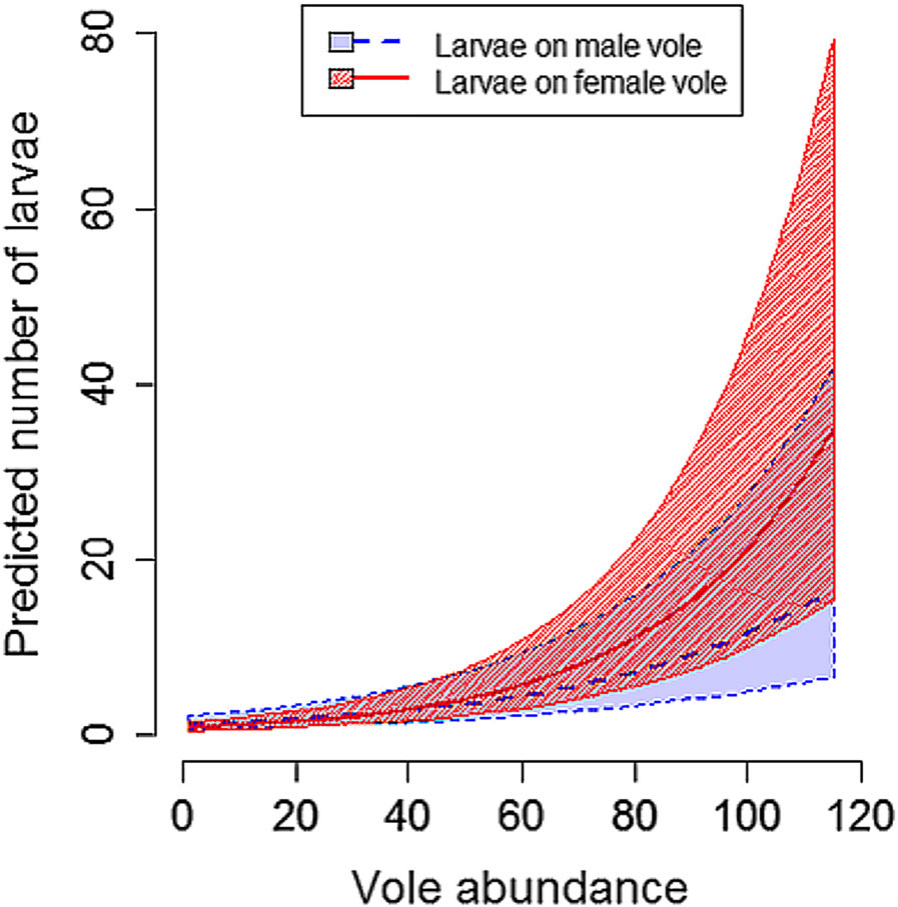
Predicted number ± standard error (SE) of larvae per bank vole (male and female separated) by vole abundance. Predictions are based on GLMM shown in Table 3

Models revealed a clear seasonal pattern in the infestation burden of *I. ricinus* larvae on bank voles (Table 3, Fig. 4): larval infestation underwent seasonality, with a peak in June and a trough in August-October. The highest infestation level was in 2013 and the lowest in 2014. In addition, bank vole infestation load with *I. ricinus* nymphs underwent seasonal variation, with a peak in May, but was stable between years (Table 4, Fig. 3).

**Table 3:** Selected best model for *I. ricinus* larvae infestation load on an individual bank vole with estimated coefficients (in log scale) explained by month (from May to October, with May as a reference), year (from 2012 to 2015, with 2012 as a reference), sex (female as a reference), body mass in grams (centred values), presence of *I. trianguliceps* females and nymphs, presence of *I. ricinus* nymphs, vole abundance during the same session, questing larvae in vegetation during the same session, the interaction between centred body mass and sex and the interaction between sex and vole abundance. We defined site and individual nested in site as nested random structure

	Estimate (SE)	*z*-value	*P*-value
Intercept	-0.923 (0.318)	-2.91	0.004
June	0.477 (0.243)	1.96	0.050
July	-0.691 (0.277)	-2.49	0.013
August	-0.900 (0.342)	-2.63	0.009
September	-1.734 (0.413)	-4.20	<0.005
October	-2.768 (0.376)	-7.36	<0.005
2013	0.720 (0.150)	4.79	<0.005
2014	-0.688 (0.275)	-2.50	0.012
2015	-0.248 (0.169)	-1.47	0.142
Male	0.996 (0.219)	4.55	<0.005
Body mass	0.020 (0.010)	2.03	0.043
Presence of *I. trianguliceps* female	0.402 (0.154)	2.61	0.009
Presence of *I. trianguliceps* nymphs	0.202 (0.101)	2.00	0.046
Presence of *I. ricinus* nymphs	0.526 (0.132)	3.97	<0.005
Vole abundance	0.033 (0.005)	6.43	<0.005
Amount of questing larvae during the same session	0.027 (0.009)	2.86	0.004
Interaction: Sex(Male)*Body mass	0.048 (0.016)	3.02	0.003
Interaction: Sex (Male)*Vole abundance	-0.009 (0.003)	-3.20	0.001
Random effects			
Site	σ^2^ = 0.06 (SD = 0.25)		
Individual nested in site	σ^2^ = 0.22 (SD = 0.47)		
Negative binomial dispersion parameter	1.70 (SE = 0.24)		

σ^2^ is the variance attributable to random effect. Number of observations: Total = 1007; Site = 4, Site:Individual = 658

*Abbreviations*: *SD* standard deviation, *SE* standard error

**Table 4:** Selected best model for *I. ricinus* nymph infestation load on an individual bank vole with estimated coefficients (in log scale) explained by month (from May to October, with May as reference), sex (female as reference), presence of *I. trianguliceps* larvae and females and presence of *I. ricinus* larvae, centered body mass and its squared value. We defined site and individual nested in site as nested random structure

	Estimate (SE)	*z*-value	*P*-value
Intercept	-2.994 (0.617)	-4.86	<0.005
June	-1.325 (0.385)	-3.44	<0.005
July	-1.360 (0.429)	-3.17	0.002
August	-2.103 (0.518)	-4.06	<0.005
September	-3.043 (0.643)	-4.73	<0.005
October	-2.956 (0.732)	-4.04	<0.005
Male	1.787 (0.298)	6.00	<0.005
Body mass	0.219 (0.036)	6.14	<0.005
Body mass^2^	-0.009 (0.003)	-2.80	0.005
Presence of *I. trianguliceps* larvae	0.709 (0.247)	2.87	0.004
Presence of *I. trianguliceps* female	1.012 (0.318)	3.18	0.002
Vole abundance	0.014 (0.006)	2.30	0.021
Random effects			
Site	σ^2^ = 0.75 (SD = 0.87)		
Individual nested in site	σ^2^ = 0.01 (SD = 0.09)		
Negative binomial dispersion parameter	1.00 (SE = 0.46)		

σ^2^ is the variance attributable to random effect. Number of observations: Total = 1,007; Site = 4; Site:Individual = 658

*Abbreviations*: *SD* standard deviation, *SE* standard error

For any given month, nymph infestation on voles was positively associated with bank vole abundance (Table 4). Similarly, larval infestation level increased with bank vole abundance, but the increase was more pronounced among female bank voles than among males (Table 3, Fig. 4). Moreover, infestation with larvae was positively associated with the amount of questing larvae observed in the environment (Table 3), whereas the bank vole infestation load with nymphs was not associated with the amount of questing nymph (i.e. the abundance of questing nymphs was not selected in the best model, Additional file 1: Table S5).

Tick infestation intensity on a host varied with individual characteristics such as age, sex and co-infestation. The oldest male bank voles (i.e. those with highest body mass) were the most intensely infested with larvae (Table 3). Moreover, bank vole infestation load with *I. ricinus* larvae was positively associated with co-infesting *I. trianguliceps* females and nymphs and *I. ricinus* nymphs (Table 3), whereas the infestation with *I. ricinus* nymphs increased with the presence of *I. trianguliceps* larvae and females (Table 4). In addition, the infestation load with nymphs showed a non-linear relationship with body mass: infestation load increased until voles reached 32.4 g, whereupon any further increase in body mass led to a reduction of the infestation burden (Table 4).

## Discussion

In this study, we characterized the temporal dynamics of *I. ricinus* by assessing its abundance in the vegetation and its infestation load in one of its main host in the northernmost part of its range. We focused on bank voles, which parasitism provides insightful information concerning the local immature tick communities. Moreover, we identified risk periods - when humans are likely to encounter tick bites - in boreal forests and seasonal patterns that might be relevant for tick-borne pathogen circulation.

### Tick seasonality

We identified that the highest tick abundance was in early summer (May-June) and early autumn (August-September), which are consequently the periods of increased risk for humans to encounter *I. ricinus* ticks in boreal forests. The same pattern of bimodal questing activity was previously found in southern Finland for nymph and adult ticks in coniferous and deciduous forests, whereas larvae showed a bimodal occurrence with a larger peak in September than in June [14]. Overall, two types of tick questing activity patterns have been described in Europe: in highly seasonal climates, such as those in central Europe, a bimodal questing activity with early spring and autumn peaks has been described for all life stages of *I. ricinus* [43]. However, in milder climates, with less climatic variation between seasons, only one peak of activity was observed for all life stages; in either spring or early summer [43]. In the present study, nymphs and adults showed bimodal activity, whereas larvae showed a unimodal activity pattern. This unimodal activity pattern could arise from egg production during the preceding year, the product of which overwintered as eggs or as larvae [44] or from egg production during the same spring. It could be argued that the inclusion of a year*month interaction term in the model would have captured between year seasonal variations suggested by the raw data (Additional file 1: Figure S1), and would have revealed both unimodal and bimodal activity patterns for larvae. However, data from a longer time series would be needed in order to clarify this point.

Our results seem to indicate the coexistence of two age cohorts of ticks during the same year. Larvae detected on bank voles and in the vegetation in early summer become nymphs in autumn, which can exhibit two different behaviors: immediate questing behavior in autumn; or activity postponed until the next spring after a behavioral diapause [45–47]. In our study, the largest peak of nymphal activity was observed in spring, suggesting that the second behavior was predominant [45]. In addition, we found a peak in questing adults 2 years after the largest larval infestation, indicating a probable 2-year period between larvae blood meal and adults, and suggesting a 2 to 3 year life-cycle from egg to adult for *I. ricinus* in our study area. Furthermore, in an additional model, the amount of questing nymphs was explained, amongst others explanatory variables, by the total amount of larvae that fed on bank vole the year before (GLMM negative binomial: *estimate* (*± SE*) = 0.005 ± 0.002, *P* = 0.0071, see Additional file 1: Tables S6 and S7). This model confirmed firstly, that the variation in bank vole larval infestation was translated into nymph abundance and secondly, a 1 year delayed relationship between bank vole larval infestation and questing nymphs.

We observed an effect of saturation deficit on larval and adult questing behavior, but not on nymphs, as described in other studies [40, 45]. Ticks respond to microclimate, but climatic variations measured in this study presumably reflect only roughly microclimatic variations and could explain the lack of association between saturation deficit and nymph activity found in our study. On the other hand, nymphs might also be acclimatized to local conditions and therefore their questing behavior may vary compared to nymphs studied in other locations [48, 49]. This is further supported by the optimal saturation deficit value of 3.16 mm Hg over which the adult questing activity decreased, when an optimum of 4.4 mm Hg has been previously noted elsewhere [9].

Our ratio larvae:nymph:adult was 3.5:3.3:2.0 and was therefore different from theoretical biological expectation (100:10:2) [30], indicating a possible underestimation of nymphs, and particularly of larvae, which show an important patchiness in distribution. Indeed, the blanket dragging technique is limited by variability in sampling efficiency given the nature of the substrate, the wind speed during sampling, and the height, type and growth stage of the vegetation [50, 51]. Moreover, the total tick population is not accessible by flagging given that diapausing ticks, parasitizing ticks, quiescent ticks or rehydrating individual are not questing in vegetation. Associating bank vole screening to the blanket dragging provided a broader view of immature ticks’ population by beneficiating from the buffer effect that the host offers against the larvae patchiness and the drop of activity in case of unfavourable microclimate [32]. The two approaches are complementary and had led to similar results supporting further the idea that bank voles play an important role as host for immature ticks in the area.

### Dynamics of tick in immature stages and bank vole population are related

Questing and parasitizing abundances of larvae and nymphs showed positive associations with bank vole abundance during a given session. Regarding nymphs, this positive relationship might arise from better engorgement success for larvae in high bank vole abundance. However, regarding larvae, this correlation does not imply a causative relationship since bank voles do not contribute to larvae production, which relies on large mammal availability [52]. Consequently, this positive relationship between the abundance of larvae and bank voles might reflect large-mammal density variations or might reveal a functional response: larvae may increase questing behavior in response to increased chemical signals produced by large bank vole populations [53]. This hypothesis requires further attention and needs to be experimentally quantified. Additionally, abundance of other species known to host adult stages needs to be quantified.

The largest burden of nymph parasitism in voles was observed in May, whereas peaks of questing nymphs in the vegetation were observed in May-June and September. In May, vole populations are mainly composed of overwintered sexually active adults; highly mobile males exhibit large home ranges in their search for receptive females [54]. Therefore, the probability of encountering questing nymphs present at a low level in the recovering spring vegetation is increased [55]. In September, bank vole contact rate with nymphs might be lower due to taller vegetation, which allows nymphs to quest higher on plants, where they can contact larger mammal hosts [47]. Moreover, the bank vole develops an acquired resistance to ticks, leading to a significant reduction of infestation success after the first infestation [56, 57], which could lead to poor infestation success during the second nymph peak in September. However, our data (Table 3) provide little support for this hypothesis as regards larvae infestation that increases with animal weight, which is used here as a proxy for age, when a decrease in the relationship was expected under acquired immunity hypothesis. As a consequence, the main period for larval and nymph co-infestation on bank voles is in early summer. The epidemiological consequences of these co-infestations are discussed below. Concerning larvae, we identified an infestation peak in June, which is in accordance with the peak of larvae questing activity and in accordance with previous surveys [58].

Male bank voles were more commonly infested with nymphs than females and the infestation increased with bank vole abundance. This sex-specific infestation load has been described previously [59] and may not only be due to the immunosuppressing role of testosterone [60–62], but also to sex-specific behavioral differences, e.g. in home range sizes [63]. Surprisingly, we found larvae infestation differs with population density; females carried more larvae at high population density, whereas males carried more nymphs at any population density. A different use of vertical space by bank vole males and females in high population densities can be hypothesized, leading males to come into contact with more nymphs that quest higher in vegetation, whereas females, which exhibit aggressive defensive behavior against intruders during the reproductive season [64], would stay close to the ground, i.e. at larvae level. More attention should be paid to the use of vertical space by bank voles in order to clarify the potential role of vertical space use causing differences between individuals in their tick infestation load.

Our data show a concomitant early summer questing activity between larvae and nymphs, and a parasitic aggregation between larvae and nymphs of *I. ricinus* on bank voles*,* which are relevant from an epidemiological point of view. The simultaneous activity of larvae and potentially infected nymphs occurs when rapidly rising temperatures in spring allow the simultaneous emergence of larvae and nymphs from overwintering diapause. In these conditions, pathogen transmission from infected nymphs to susceptible larvae can occur *via* simultaneous feeding on the same host, even without systemic infection of the host. This co-feeding transmission pathway is important for several zoonotic tick-borne pathogens, especially those with short-lived or non-systemic infections in the rodent host, such as *Anaplasma phagocytophilum* or tick-borne encephalitis virus (TBEV), respectively [65–68].

### Synchronous infestations on bank voles

In addition to aggregation between *I. ricinus* life stages on bank voles, we found a significant aggregation between tick species, with *I. ricinus* infestation load increasing with the presence of *I. trianguliceps. I. trianguliceps* is a nidicolous species associated with rodents and insectivores, which does not quest in the vegetation and hence does not come into contact with humans [69]. Even if it is not involved in zoonotic transmission, *I. trianguliceps* is responsible for maintaining the enzootic cycle of potential zoonotic pathogens such as *Anaplasma phagocytophilum* [70, 71] or *Babesia microti* [72, 73]. Both of these pathogens have been identified in Finnish bank voles [74]. *Ixodes trianguliceps* could contribute to the sylvatic cycle of pathogens that the generalist *I. ricinus* could transmit to humans, who are considered as dead-end hosts. Hence, the between-species ectoparasite aggregation is also relevant from an epidemiological point of view.

## Conclusion

In northern European urban forests, population dynamics of bank voles and questing *I. ricinus* larvae and nymphs are related, suggesting higher tick abundance and consequently higher risk of tick-borne pathogens for human during the rodent population peak. Larvae and nymphs showed synchronous activity, which increases the transmission opportunity for several pathogens and which are the prerequisite conditions for the maintenance of some pathogens such as TBEV. Further studies should focus on assessing the prevalence of tick-borne pathogens in the bank vole and in questing ticks in order to specify the zoonotic risk. Recent models demonstrate a dampening of vole population cycles in northern Europe [75], which could therefore be translated into the population dynamics of ticks.

## Additional file

Additional file 1: Figure S1. Average monthly saturation deficit and temperature during the monitoring years. **Figure S2.** Observed mean abundance of ticks in vegetation per session, from May 2012 to October 2015. **Figure S3.** Mean number of vole captured per trap-night at each session and in each site, from May 2012 to October 2015. **Table S1.** Selection table for models explaining the abundance of ticks questing in the vegetation. **Figure S4.** Predicted number of larvae, nymphs and pooled nymphs and females per 100 m2 of vegetation explained by bank vole abundance. **Table S2.** Selection table for models explaining the abundance of ticks questing in the vegetation. **Table S3.** Total number of ticks (per species and stage) collected on voles. **Figure S5.** Vole infestation per session with *I. ricinus* larvae and nymphs from May 2012 to October 2015. **Table S4.** Selection table for models explaining the abundance of infesting larvae. **Table S5.** Selection table for models explaining the abundance of infesting nymphs. **Table S6.** Additional model for the abundance of nymphs questing in the vegetation. **Table S7.** Selection table for the additional model explaining the abundance of questing nymphs. (DOCX 3706 kb)
