# Supplementary material for: Temporal dynamics of the tick Ixodes ricinus in northern Europe: epidemiological implications
Source: Parasit Vectors. 2017 Mar 31;10:166. doi: 10.1186/s13071-017-2112-x (PMC5374616; doi:10.1186/s13071-017-2112-x)
Supplement: Additional file 1: Figure S1. — Average monthly saturation deficit and temperature during the monitoring years. Figure S2. Observed mean abundance of ticks in vegetation per session, from May 2012 to October 2015. Figure S3. Mean number of vole captured per trap-night at each session and in each site, from May 2012 to October 2015. Table S1. Selection table for models explaining the abundance of ticks questing in the vegetation. Figure S4. Predicted number of larvae, nymphs and pooled nymphs and females per 100 m2 of vegetation explained by bank vole abundance. Table S2. Selection table for models explaining the abundance of ticks questing in the vegetation. Table S3. Total number of ticks (per species and stage) collected on voles. Figure S5. Vole infestation per session with I. ricinus larvae and nymphs from May 2012 to October 2015. Table S4. Selection table for models explaining the abundance of infesting larvae. Table S5. Selection table for models explaining the abundance of infesting nymphs. Table S6. Additional model for the abundance of nymphs questing in the vegetation. Table S7. Selection table for the additional model explaining the abundance of questing nymphs. (DOCX 3706 kb) [file 13071_2017_2112_MOESM1_ESM.docx]

**Additional file 1**

**Content: Figure S1.** Average (± SE) monthly saturation deficit and temperature during the monitoring years, measured in the weather station. **Figure S2.** Observed mean abundance of ticks in vegetation per session, from May 2012 to October 2015 (NB: in 2014 ticks were not dragged in October, due to poor weather conditions).**Figure S3.** Mean number of vole captured per trap-night at each session and in each site, from May 2012 to October 2015. **Table S1.** Model selection table for the abundance of ticks questing in the vegetation explained by vole abundance (MNA), month, year, abundance in other stages during the same session (nymph (Ny), adult (Ad), larva (Larv)), abundance of ticks in a previous life-stage collected during the previous session (lag(Ad), Lag(Ny), Lag(Larv)), total amount of larvae that fed on voles during the previous year (LagY(Larv)), total amount of larvae that fed on bank vole during the same early summer (May and June) (LagS(Larv)). **Figure S4.** Predicted number (± SE) of larvae, nymphs and pooled nymphs and females per 100 m2 of vegetation explained by bank vole abundance. Predictions are based on GLMM showed in Table 1. **Table S2.** Model selection table for the abundance of ticks questing in the vegetation, explained by vole abundance (MNA), Saturation deficit (SatDef) and Saturation deficit2 (SatDef2), abundance in other stages during the same session (adult (Ad), nymph (Ny), larva(Larv)), abundance of ticks in a previous life-stage collected during the previous session (lag(Ad), Lag(Ny), Lag(Larv)). **Table S3.** Total number of ticks (per species and stage) collected on voles, with the minimum and maximum tick infestation per vole, the percentage of vole infested with a particular tick stage or species, the mean number of ticks infesting a vole and the mean number of ticks per infested vole. **Figure S5.** Vole infestation per session (± SE) with *I. ricinus* larvae and nymphs from May 2012 to October 2015. **Table S4.** Model selection table for the abundance of infesting larvae, explained by month, year, bank vole sex, centered body mass (cBm) and cBm2, infestation with ticks in other species or other stages (ItL (*I. trianguliceps* larvae), ItF (*I. trianguliceps* female), ItN (*I. trianguliceps* nymph), IrN (*I. ricinus* nymph)) and with fleas, abundance of questing larvae (Larv), vole abundance (MNA), and the interaction between sex and vole abundance and the interaction between sex and body mass. **Table S5.** Model selection table for the abundance of infesting nymphs, explained by month, year, bank vole sex, centered body mass (cBm) and cBm2, infestation with ticks in other stages or other species (ItL (*I. trianguliceps* larvae), ItF (*I. trianguliceps* female), ItN (*I. trianguliceps* nymph), IrL (*I. ricinus* larvae)) and with fleas, abundance of questing nymphs (Nymph), vole abundance (MNA), the interaction between sex and vole abundance and the interaction between sex and body mass. **Table S6.** Additional model for the abundance of nymphs questing in the vegetation. **Table S7.** Model selection table concerning the abundance of questing nymphs, explained by month, year, vole abundance (MNA), the total amount of larvae that fed on voles the year before (LagY(Larv)), the total amount of larvae that fed on voles during the same summer (May and June) (LagS(Larv)), the amount of larvae (Larv) and adult (Ad) in vegetation during the same session

**List of variables in the datasets associated with this manuscript:** Date; Month; Year; Session: trapping period within a month; Av_temp, Av_hum, Av_baro: daily average temperature, humidity, atmospheric pressure recorded at the meteorological station of Nenäinniemi; Vole_MNA_general: minimum number of voles alive during the session; areas: location of the sampling areas: 1= Kylmänoro, 2= Sippulanniemi, 3= Hämeenlahti, 4= Jyskänlaakso; ticks: presence/absence of tick in vegetation during the session; irl, irn, irf, irm, irnf, ad, irtot: total number of *I. ricinus* larvae, nymphs, females, males, females and nymphs, males and females, all stages pooled together collected with the flagging method; it: number *of I.trianguliceps* all stages pooled together collected with the flagging method; m: number of meters flagged; satdef: mean saturation deficit during the sampling day; trap: vole trap location; ind: vole identification number; sex: vole sex (1: female, 2: male); weight: vole weight (g); head: vole head width (mm); ticks: presence/absence of ticks on the vole; fleas: presence/absence of fleas on the vole; IrL_TOT, IrN_TOT, IrF, Ir_TOT: total number of *I. ricinus* larvae, nymphs, females and all stages pooled infesting an individual vole; IrL_PA, IrN_PA, Ir_PA: presence/absence of larvae, nymphs and all stages of *I. ricinus* infesting an individual vole; ItL_TOT, ItN_TOT, ItF_TOT, M_It, It_TOT: total number of *I. trianguliceps* larvae, nymphs, females, males, and all stages pooled infesting an individual vole; ItL_PA, ItN_PA, ItF_PA, It_PA: presence/absence of *I.trianguliceps* larvae, nymphs, females and all stages pooled infesting an individual vole, NoN.identified.ticks: total number of ticks non identified at species level sampled from an individual vole; Tot_Ticks: total number of ticks all stages and all species pooled together infesting an individual vole; irl_100, irn_100, irf_100, irm_100, irnf_100, irtot_100: total number of *I.ricinus* larvae, nymphs, females, males, nymphs and females, all stages pooled together flagged in 100m2 of vegetation during a session; it_100: total number of *I.trianguliceps* flagged in 100m2 of vegetation during session


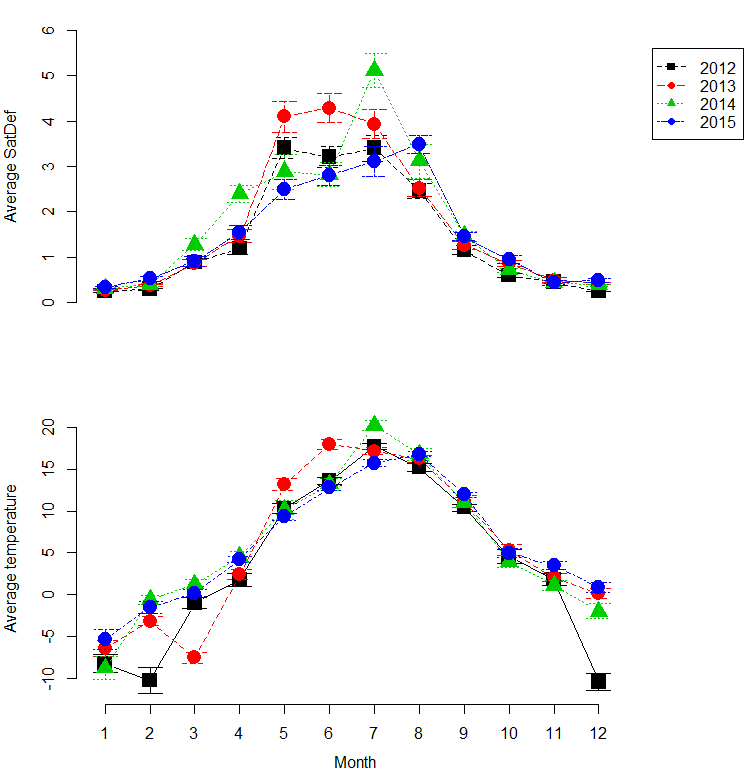


**Figure S1. Average (± SE) monthly saturation deficit and temperature during the monitoring years, measured in the weather station**


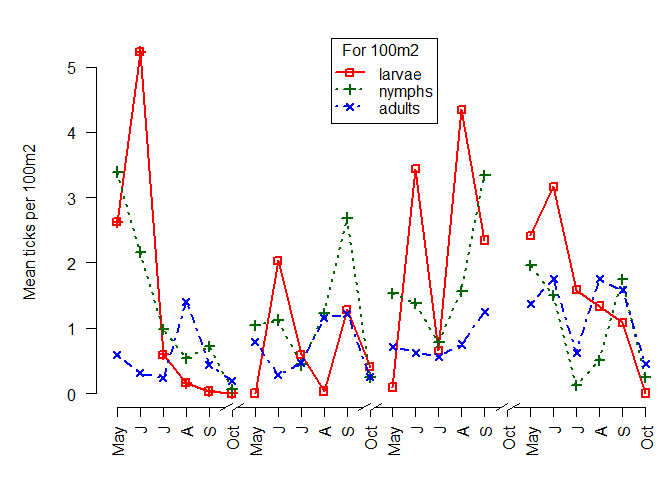


**Figure S2. Observed mean abundance of ticks in vegetation per session, from May 2012 to October 2015 (NB: in 2014 ticks were not dragged in October, due to poor weather conditions).**


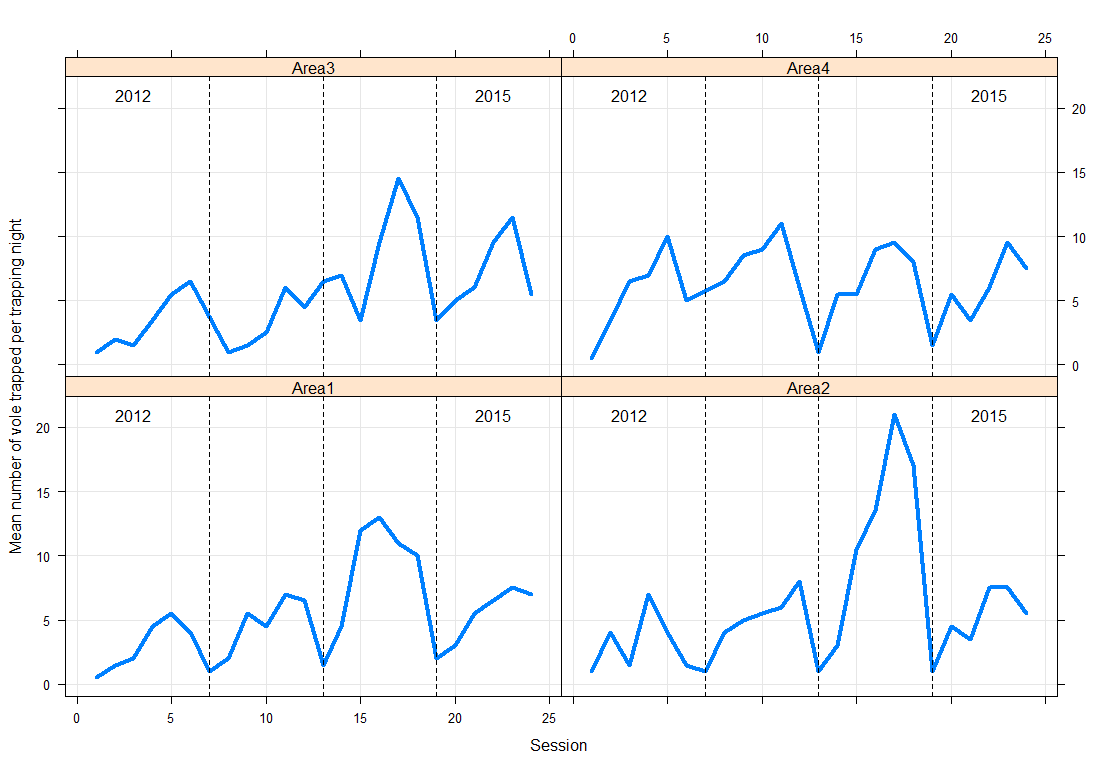


**Figure S3. Mean number of vole captured per trap-night at each session and in each site, from May 2012 to October 2015.**

**Table S1. Model selection table for models showed in Table 1. The abundance of ticks questing in the vegetation was explained by vole abundance (MNA), month, year, abundance in other stages during the same session (nymph (Ny), adult (Ad), larva (Larv)), abundance of ticks in a previous life-stage collected during the previous session (lag(Ad), Lag(Ny), Lag(Larv)), total amount of larvae that fed on voles during the previous year (LagY(Larv)), total amount of larvae that fed on bank vole during the same early summer (May and June) (LagS(Larv)). Full model and all models laying at 2 AICc difference from the lowest AICc are showed with their degree of freedom (Df).**

| Questing larvae | Df | AICc | Delta |
| --- | --- | --- | --- |
| FULL Lag(Ad)+ Ny+Ad+Year+Month+MNA  Best Month+MNA  Month+Ny+MNA  Month+MNA+lag(Ad)  Month+Ny  Ad+MNA+Month+Ny  Ad+MNA+Month | 15  9  10  10  9  11  10 | 491.8  484.1  484.4  485.3  485.4  485.5  485.8 | 7.73  0.00  0.30  1.18  1.30  1.40  1.73 |
| Questing nymphs |  |  |  |
| FULL Lag(Larv)+Larv+Ad+Year+Month+MNA  Best Month+MNA  Lag(Larv)+Month+MNA | 15  9  10 | 521.8  510.8  510.1 | 11.71  0.71  0.00 |
| Questing adults |  |  |  |
| FULL Lag(Ny)+Larv+Ny+Year+Month+MNA  Best Month+Year  Larv+Month+Year  Larv+Month+Year+Ny  Month+Ny+Year  Larv+MNA+Month+Year | 15  11  12  13  12  13 | 451.8  447.0  446.4  446.5  447.5  448.3 | 5.34  0.58  0.00  0.11  1.09  1.91 |
| Questing (female+nymph) |  |  |  |
| FULL Month+Year+MNA  Best Month+MNA | 12  9 | 552.0  546.0 | 6.04  0.00 |


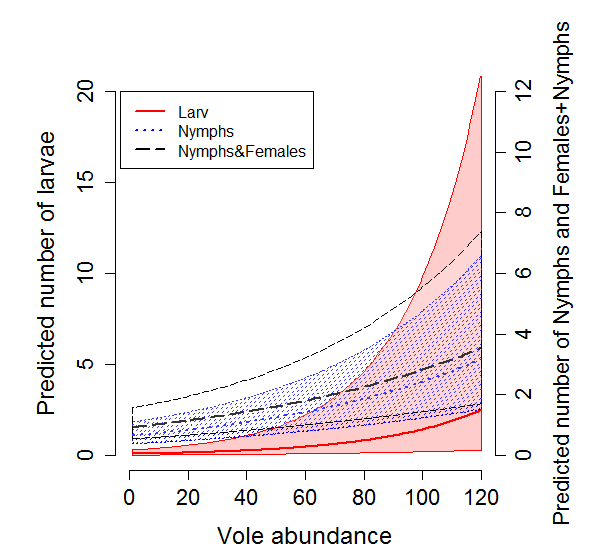


**Figure S4. Predicted number** (± **SE)** **of larvae, nymphs and pooled nymphs and females per 100 m^2^ of vegetation explained by bank vole abundance. Predictions are based on GLMM showed in Table 1.**

**Table S2. Model selection table for models showed in Table 2. The abundance of ticks questing in the vegetation was explained by vole abundance (MNA), Saturation deficit (SatDef) and Saturation deficit^2^ (SatDef^2^), abundance in other stages during the same session (adult (Ad), nymph (Ny), larva(Larv)), abundance of ticks in a previous life-stage collected during the previous session (lag(Ad), Lag(Ny), Lag(Larv)). Full model and all models laying at 2 AICc difference from the lowest AICc are showed with their degree of freedom (Df).**

| Questing larvae | Df | AICc | Delta |
| --- | --- | --- | --- |
| FULL Lag(Ad)+SatDef+SatDef²+MNA+Ny+Ad+Year  Best Lag(Ad)+SatDef+MNA  Lag(Ad)+SatDef+SatDef²+MNA  Lag(Ad)+SatDef+SatDef²+MNA+Ny  Lag(Ad)+SatDef+ MNA+Ny | 12  6  7  8  7 | 485.6  477.10  477.1  477.6  478.6 | 8.62  0.00  0.07  0.64  1.55 |
| Questing nymphs |  |  |  |
| FULL Lag(Larv)+SatDef+SatDef²+MNA+Larv+Ad+Year  Best Ad  Ad+SatDef+MNA  Ad+Larv  Ad+MNA  Ad+MNA+Larv  Ad+SatDef²+MNA  Ad+Larv+SatDef+MNA  Ad+Larv+Year  SatDef²+SatDef+MNA+Ad  SatDef²+ MNA+Ad+Larv  Ad+Year | 12  4  6  5  5  6  6  7  8  7  7  7 | 565.5  555.8  555.1  555.4  555.9  556.0  556.0  556.2  556.8  556.8  556.9  557.0 | 10.35  0.69  0.00  0.25  0.77  0.83  0.89  1.05  1.66  1.67  1.77  1.89 |
| Questing adults |  |  |  |
| FULL Lag(Ny)+SatDef+SatDef²+MNA+Larv+Ny+Year  Best Satdef+SatDef²+Ny+Lag(Ny)+Year  Satdef+SatDef²+Ny+Lag(Ny)+Year+Larv  Satdef+SatDef²+Ny+Lag(Ny)+Year+MNA | 12  10  11  11 | 459.0  458.9  458.5  460.0 | 0.47  0.37  0.00  1.48 |
| Questing (female+nymph) |  |  |  |
| FULL SatDef+SatDef²+MNA+Year  Best MNA  Satdef+Satdef²+MNA  Year  Satdef+MNA  MNA+Year | 9  4  6  6  5  7 | 603.7  600.3  600.0  600.1  601.3  601.8 | 3.70  0.31  0.00  0.04  1.25  1.72 |

Table S3. Total number of ticks (per species and stage) collected on voles, with the minimum and maximum tick infestation per vole, the percentage of vole infested with a particular tick stage or species, the mean number of ticks infesting a vole and the mean number of ticks per infested vole (SE=standard error, N=1007 observations).

|  | Range | Total number | % Vole infested | Mean per vole (SE) | Mean per vole infested by the tick stage and species (SE) |
| --- | --- | --- | --- | --- | --- |
| ***I. ricinus*** | | | | | |
| Larvae | [0; 46] | 2290 | 59.19 | 2.27 (0.13) | 3.84 (0.20) |
| Nymph | [0; 13] | 178 | 9.83 | 0.18 (0.03) | 1.80 (0.04) |
| Female | [0; 1] | 1 | 0.099 | - | - |
| Total | [0; 50] | 2469 | 61.17 | 2.45 (0.15) | 4 (0.22) |
| ***I. trianguliceps*** | | | | | |
| Larvae | [0; 27] | 718 | 28.40 | 0.71 (0.06) | 2.51 (0.17) |
| Nymph | [0; 8] | 275 | 18.47 | 0.27 (0.02) | 1.48 (0.08) |
| Female | [0; 4] | 84 | 5.86 | 0.08 (0.012) | 1.42 (0.10) |
| Male | [0; 2] | 4 | 0.40 | 0.004 (0.002) | 1.33 (0.33) |
| Total | [0;27] | 1081 | 42.9 | 1.07 (0.07) | 2.50 (0.13) |


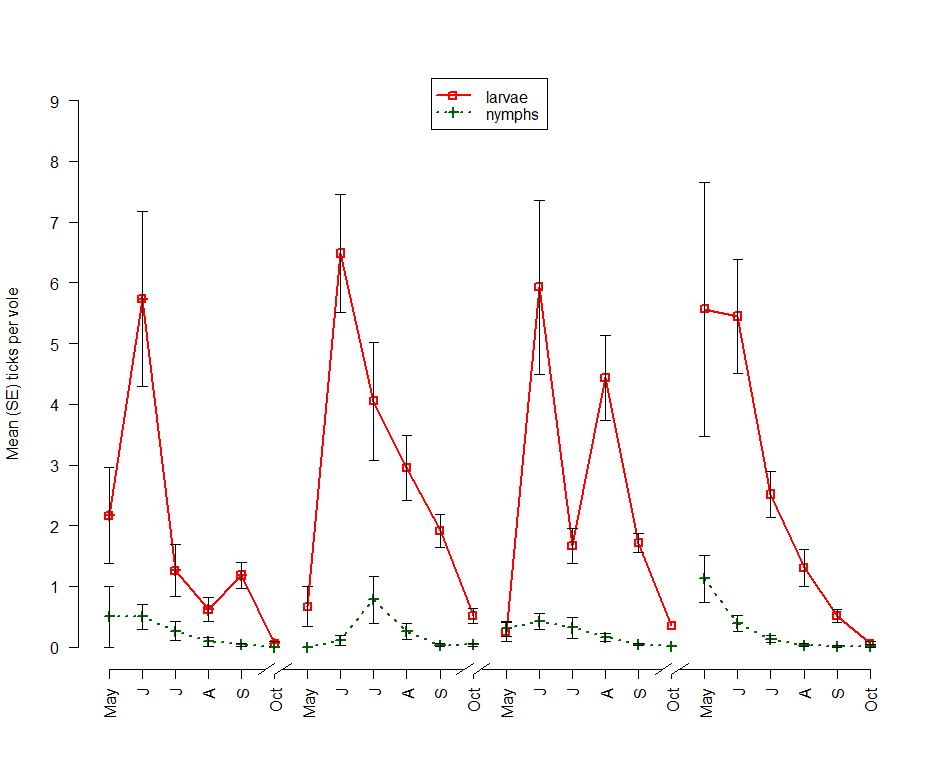


**Figure S5. Vole infestation per session (± SE) with *I. ricinus* larvae and nymphs from May 2012 to October 2015**

**Table S4. Model selection table for models showed in Table 3. The abundance of infesting larvae was explained by month, year, bank vole sex, centered body mass (cBm) and cBm^2^, infestation with ticks in other species or other stages (ItL (*I. trianguliceps* larvae), ItF (*I. trianguliceps* female), ItN (*I. trianguliceps* nymph), IrN (*I. ricinus* nymph)) and with fleas, abundance of questing larvae (Larv), vole abundance (MNA), and the interaction between sex and vole abundance and the interaction between sex and body mass**

| **Model** | AIC | Df |
| --- | --- | --- |
| Full Model:  Month + Year + Sex + cBm + cBm² + Sex *cBm + ItL + ItF + ItN + IrN + MNA + Larv + Fleas + Sex*MNA | 3469.0 | 19 |
| Month + Year + Sex + cBm + cBm² + Sex *cBm + ItL + ItF + ItN + IrN + MNA + Larv + Sex*MNA | 3467.0 | 18 |
| Month + Year + Sex + cBm + Sex *cBm + ItL + ItF + ItN + IrN + MNA + Larv + Sex*MNA | 3465.1 | 17 |
| Month + Year + Sex + cBm + Sex *cBm + ItF + ItN + IrN + MNA + Larv + Sex*MNA | 3464.0 | 16 |

**Table S5. Model selection table for models showed in Table 4. The abundance of infesting nymphs was explained by month, year, bank vole sex, centered body mass (cBm) and cBm^2^, infestation with ticks in other stages or other species (ItL (*I. trianguliceps* larvae), ItF (*I. trianguliceps* female), ItN (*I. trianguliceps* nymph), IrL (*I. ricinus* larvae)) and with fleas, abundance of questing nymphs (Nymph), vole abundance (MNA), the interaction between sex and vole abundance and the interaction between sex and body mass**

| **Model** | AIC | Df |
| --- | --- | --- |
| Full model:  Month + Year + Sex + cBm + cBM^2^ + ItL + ItN + ItF + IrL + Sex * Bm + Sex * MNA + MNA + Nymph + Fleas | 708.55 | 19 |
| Month + Sex + cBm + cBM^2^ + ItL + ItN + ItF + IrL + Sex * Bm + Sex * MNA + MNA + Nymph + Fleas | 702.82 | 16 |
| Month + Sex + cBm + cBM^2^ + ItL + ItN + ItF + IrL + Sex * Bm + Sex * MNA + MNA + Nymph | 700.83 | 15 |
| Month + Sex + cBm + cBM^2^ + ItL + ItF + IrL + Sex * Bm + Sex * MNA + MNA + Nymph | 698.93 | 14 |
| Month + Sex + cBm + cBM^2^ + ItL + ItF + IrL + Sex * Bm + Sex * MNA + MNA | 697.15 | 13 |
| Month + Sex + cBm + cBM^2^ + ItL + ItF + IrL + Sex * Bm + MNA | 695.96 | 12 |
| Month + Sex + cBm + cBM^2^ + ItL + ItF + Sex * Bm + MNA | 695.16 | 11 |
| Month + Sex + cBm + cBM^2^ + ItL + ItF + MNA | 695.57 | 10 |

**Table S6. Additional model for the abundance of nymphs questing in the vegetation**

| ***Y = Nymph abundance in vegetation*** | Estimate(SE) | z-value | p-value |
| --- | --- | --- | --- |
| Intercept | -0.108(0.28) | -0.38 | 0.7028 |
| 2014 | -0.709(0.38) | -1.85 | 0.0637 |
| 2015 | -1.051(0.40) | -2.62 | **0.0089** |
| June | -0.542(0.28) | -1.96 | **0.0498** |
| July | -2.132(0.40) | -5.29 | **<0.005** |
| August | -1.446(0.49) | -2.94 | **<0.005** |
| September | -0.924(0.62) | -1.50 | 0.1331 |
| October | -2.802(0.54) | -5.23 | **<0.005** |
| Tot amount of larvae that fed on voles the year before | 0.005(0.002) | 2.69 | **0.0071** |
| Amount of tick larvae questing at the same session | -0.078(0.03) | -2.69 | **0.007** |
| Vole abundance | 0.021(0.008) | 2.55 | **0.0109** |
| Random effect: site | σ2 = 0.1161 (SD =0.34) | | |
| Negative binomial dispersion parameter | 5.6955 (SE = 1.80) | | |
| AIC | 390.2 | | |

Additional model for the abundance of *I. ricinus* nymphs questing in the vegetation with estimated coefficients (in log scale), explained by month (from May to October, with May as a reference), year (from 2013 to 2015, with 2013 as a reference), the total amount of larvae that fed on voles the year before, the amount of larvae in vegetation during the current session and the vole abundance. σ^2^ is the variance attributable to random effect. Number of observations: total = 68, Site = 4

**Table S7. Model selection table concerning the model showed in table S6. The abundance of questing nymphs was explained by month, year, vole abundance (MNA), the total amount of larvae that fed on voles the year before (LagY(Larv)), LagS(Larv): the total amount of larvae that fed on voles during the same summer (May and June), the amount of larvae (Larv) and adults (Ad) in vegetation during the same session**

| Questing nymphs (2) | Df | AIC | Δ |
| --- | --- | --- | --- |
| FULL Year+Month+MNA+LagY(Larv)+LagS(Larv)+ Larv+Ad  Best Month+MNA  Larv+Month+MNA  **Larv+Year+Month+MNA+LagY(Larv)**  Larv+MNA+Month+ LagS(Larv) | 15  9  10  13  11 | 400.2  396.9  395.6  396.9  397.5 | 4.61  1.29  0.00  1.31  1.89 |
